# Antibody response to *Phlebotomus perniciosus* saliva in cats naturally exposed to phlebotomine sand flies is positively associated with *Leishmania* infection

**DOI:** 10.1186/s13071-019-3376-0

**Published:** 2019-03-26

**Authors:** André Pereira, José Manuel Cristóvão, Hugo Vilhena, Ângela Martins, Patrícia Cachola, Joaquim Henriques, Mónica Coimbra, Ana Catarino, Tereza Lestinova, Tatiana Spitzova, Petr Volf, Lenea Campino, Carla Maia

**Affiliations:** 1 Global Health and Tropical Medicine (GHMT), Instituto de Higiene e Medicina Tropical (IHMT), Universidade Nova de Lisboa (UNL), Lisboa, Portugal; 2 Center for Investigation Vasco da Gama (CIVG), Department of Veterinary Medicine, Vasco da Gama Universitary School, Coimbra, Portugal; 3 University Veterinary Hospital of Coimbra, Coimbra, Portugal; 4 Animal and Veterinary Research Centre (CECAV), University of Trás-os-Montes and Alto Douro, Vila Real, Portugal; 5 Hospital Veterinário da Arrábida, Azeitão, Portugal; 6 Hospital Veterinário do Algarve, Faro, Portugal; 7 Hospital Veterinário de Berna, Lisboa, Portugal; 8 Clínica Veterinária Porto Seguro, Olhão, Portugal; 9 VetCoa-Serviços Veterinários, Sabugal, Portugal; 10 Department of Parasitology, Faculty of Science, Charles University, Prague, Czech Republic

**Keywords:** Antibodies, Cat, *Leishmania infantum*, *Phlebotomus perniciosus*, Saliva, Portugal

## Abstract

**Background:**

Zoonotic leishmaniosis, caused by the protozoan *Leishmania infantum*, is a public and animal health problem in Asia, Central and South America, the Middle East and the Mediterranean Basin. Several phlebotomine sand fly species from the subgenus *Larroussius* are vectors of *L. infantum*. Data from dogs living in endemic areas of leishmaniosis advocate the use of antibody response to phlebotomine sand fly saliva as an epidemiological biomarker for monitoring vector exposure. The aim of this study was to analyse the exposure of cats to phlebotomine sand flies using detection of IgG antibodies to *Phlebotomus perniciosus* saliva. The association between phlebotomine sand fly exposure and the presence of *Leishmania* infection was also investigated.

**Results:**

IgG antibodies to *P. perniciosus* saliva were detected in 167 (47.7%) out of 350 cats; higher antibody levels were present in sera collected during the period of phlebotomine sand fly seasonal activity (OR = 19.44, 95% CI: 9.84–38.41). Cats of 12–35 months had higher antibody levels than younger ones (OR = 3.56, 95% CI: 1.39–9.16); this difference was also significant with older cats (for 36–95 months-old, OR = 9.43, 95% CI: 3.62–24.48; for older than 95 months, OR = 9.68, 95% CI: 3.92–23.91). *Leishmania* spp. DNA was detected in the blood of 24 (6.9%) cats, while antibodies to *L. infantum* were detected in three (0.9%). Only one cat was positive to *Leishmania* by both techniques. Cats presenting IgG antibodies to *P. perniciosus* had a significantly higher risk of being positive for *Leishmania* infection.

**Conclusions:**

To our knowledge, this is the first study demonstrating anti-sand fly saliva antibodies in cats. The evaluation of the contact of this animal species with the vector is important to the development of prophylactic measures directed to cats, with the aim of reducing the prevalence of infection in an endemic area. Therefore, studies evaluating whether the use of imidacloprid/flumethrin collars reduces the frequency of *P. perniciosus* bites in cats are needed. It is also important to evaluate if there is a correlation between the number of phlebotomine sand fly bites and IgG antibody levels.

## Background

Zoonotic leishmaniosis, caused by the protozoan *Leishmania infantum*, is a serious public and animal health problem in several countries of Asia, Central and South America, the Middle East and the Mediterranean Basin. Domestic dogs are the major hosts of the parasite and the main domestic reservoir hosts for human infection. Nevertheless, the number of feline leishmaniosis reports and subclinical *L. infantum* infections in cats living in endemic areas has increased in recent years [1]. In fact, there is an increasing trend to consider cats as a potential primary or secondary reservoir host of *L. infantum*, rather than being an accidental host [2]. This assumption is based on several premises, namely natural susceptibility to infection, suitability to serve as a blood source for phlebotomine sand flies, infectivity to the vector, and close contact with humans where the peridomestic and domestic transmission cycles of the parasite occur [3].

*Leishmania* parasites are transmitted by phlebotomine sand flies (Diptera: Psychodidae). During the blood meal, immunogenic components present in phlebotomine sand fly saliva are injected into the vertebrate host leading to the development of anti-saliva antibodies [4]. Data from dogs living in endemic areas of leishmaniosis caused by *L. infantum* suggest the use of antibody response to salivary antigens as an epidemiological biomarker for monitoring vector exposure [5–11]. The levels of specific IgG antibodies against phlebotomine sand fly saliva positively correlate with the number of blood-fed sand flies [6–12] and decays after the end of phlebotomine sand fly seasonal activity [7, 10].

In the Old World, *L. infantum* is transmitted by several phlebotomine sand fly species belonging to the subgenus *Larroussius*, of which *Phlebotomus perniciosus* is the principal vector in the west part of Mediterranean, including Portugal [13]. Portugal is endemic for canine leishmaniosis [14] and hypoendemic for human visceral leishmaniosis [15]. Feline leishmaniosis [16] and *L. infantum* infection in cats have been documented in Portugal [17–20]. The phlebotomine sand fly season usually lasts from May until late October [13, 21, 22].

The aim of this work was to analyse the exposure of cats to phlebotomine sand flies through the detection of antibodies to *P. perniciosus* saliva, and to assess associated risk factors. The possible association between phlebotomine sand fly exposure and the presence of *Leishmania* infection was also investigated.

## Methods

### Animals and samples

From April to December 2017, a total of 350 cats with access to the outdoors from veterinary medical centres, animal shelters and from colonies (captured under the scope of trap-neuter-return programs) from Portugal, were studied. Cats were from three continental Portuguese NUTS II (Nomenclature of Units for Territorial Statistics): Centre (Coimbra and Guarda regions; *n* = 61), Lisbon (Lisbon and Setúbal regions; *n* = 266) and the Algarve region (*n* = 23).

Peripheral blood (1–2 ml) was obtained by cephalic or jugular venipuncture from each animal and collected into EDTA and serum-separating tubes. Serum and buffy coat were obtained by centrifugation and stored at -20 °C until use in serological analyses and DNA extraction, respectively.

Whenever available, data on sex, breed, fur length, age, reproductive status, lifestyle, use of insecticides/acaricides, co-habitation with other animals, presence of concomitant diseases and of clinical signs compatible with leishmaniosis (i.e. anorexia, muscular atrophy, dermatological manifestations, exercise intolerance, fever, dyspnea, epistaxis, spleen/hepatomegaly, gingivostomatitis, gastrointestinal alterations, lameness, lymphadenopathy, lethargy, ocular manifestations, pale mucous membranes polyuria/polydipsia or weight loss) were recorded for each cat.

### *Phlebotomus perniciosus* salivary glands and detection of anti-*P. perniciosus* saliva antibodies

Salivary gland homogenate (SGH) was obtained by dissecting salivary glands from 4–6 days-old *P. perniciosus* females reared under standard conditions [23]. Groups of 20 salivary glands were pooled in 20 mM Tris buffer with 150 mM NaCl and then kept lyophilized at 4 °C until used.

Anti-*P. perniciosus* IgG was measured in all sera samples by indirect enzyme-linked immunosorbent assay (ELISA). The ELISA was performed in accordance with previous studies [7] with minor modifications. Briefly, flat-bottom microtiter plates (Nunc; VWR, Radnor, Pennsylvania, USA.) were coated with *P. perniciosus* SGH (0.2 salivary gland per well) in 20 mM carbonate-bicarbonate buffer (pH 9, 100 μl/well) and incubated overnight at 4 °C. The plates were washed with PBS + 0.05% Tween 20 (PBS-Tw) and blocked with 6% (w/v) low fat dry milk diluted in PBS-Tw at 37 °C for 60 min. Feline sera diluted 1/50 in 2% (w/v) low fat dry milk/PBS-Tw was added to the wells (100 μl/well) after washing twice with PBS-Tw. After 90 min incubation at 37 °C, the plates were washed with PBS-Tw and incubated at 37 °C for 45 min with secondary antibodies [AAI26P; Bio-Rad (AbD Serotec), Hercules, California, USA] (100 μl/well) diluted 1:5000 in PBS-Tw. Following another washing cycle, the ELISA was developed using orthophenylendiamine (P23938; Sigma-Aldrich, St. Louis, Missouri, USA) (0.5 mg/ml) in a phosphate citrate buffer (pH 5.5) with 0.001% hydrogen peroxide (30%; Merck, Darmstadt, Germany). The reaction was stopped after 5 min with 10% sulphuric acid and absorbance (OP value) was measured at 492 nm using a NanoQuant (Infinite M200 Pro; Tecan, Zürich, Switzerland). Each serum was tested in duplicate. Wells without serum (but coated with SGH) were used as blanks while sera from cats living in non-endemic countries, namely Germany and Switzerland, served as negative controls. The cut-off value was calculated by the addition of three standard deviations to the mean optical density of the control sera.

### Detection of anti-*Leishmania* IgG

Anti-*Leishmania* antibodies were determined in sera by the immunofluorescence antibody test (IFAT) as previously described [18]. Briefly, a *L. infantum* MON-1 (MCAN/PT/05/IMT-373) suspension of 10^7^ promastigotes was used as antigen and the anti-cat IgG (whole molecule)-FITC (F4262; Sigma-Aldrich) was used in a dilution of l:20. A serum sample from a seropositive cat (IFAT titre 1:1204) was used as positive control [16] while the serum sample of a cat from a non-endemic country of leishmaniosis was used as negative control. The IFAT cut-off value was established at a serum dilution of 1:64 (the same as used in the laboratory for dogs) [24].

### DNA extraction and PCR amplification

DNA was extracted from buffy coat using the High Pure PCR Template Preparation Kit (Roche Diagnostics GmbH, Mannheim, Germany) according to manufacturer’s instructions. Detection of *Leishmania* DNA was done using a nested PCR protocol with primers targeting the small subunit ribosomal RNA (*SSU* rRNA) gene [25]. A positive control containing *L. infantum* MON-1 (MHOM/PT/88/IMT318) DNA and a negative control without DNA template were included in each amplification. The DNA amplicons were resolved by conventional electrophoresis on 1.5% agarose gels stained with Green Safe Premium (Nzytech, Lisbon, Portugal), using a 100 bp DNA ladder as a molecular weight marker, then visualized under UV illumination.

### Statistical analysis

An exploratory and descriptive data analysis was conducted for the main variables of the dataset. Cats were considered infected with *Leishmania* if they tested positive for at least one of the techniques (i.e. PCR or IFAT). For the quantitative variable “age in months”, the normality and the homogeneity of variance were evaluated using Kolmogorov-Smirnov/Shapiro-Wilk tests and the Levenne test, respectively. When these prerequisites were not valid, the non-parametric Mann-Whitney test was used. To explore the associations between qualitative variables and to compare proportions a Chi-square test, the alternative Fisher’s exact test or the Freeman-Halton test was performed. Confidence intervals (95% CI) for proportions were obtained by the Wilson method. This initial approach was followed by multivariate analysis that was developed to evaluate, in an integrated way, possible factors associated with the presence of antibodies against *P. perniciosus* saliva and with the presence of *Leishmania* DNA and/or antibodies to the parasite (outcome variables). First, crude odds ratios (OR crude) and 95% CIs were obtained by a simple logistic regression model to screen the effect of each explanatory variable on the outcome variables. In a second step, explanatory variables with a *P-*value ≤ 0.2 in the univariate analysis were selected and included in the multiple logistic regression model. Finally, a backward stepwise elimination procedure was implemented, using a *P-*value ≤ 0.05 as the criterion for variables to remain in the model. The Hosmer & Lemeshow goodness-of-fit test, residual analysis and determination of the area under the receiver operating characteristic curve (ROC) were performed. All statistical analyses were conducted using IBM® SPSS® Statistics version 25.0 and OpenEpi version 3.01 software.

## Results

**Fig. 1:**
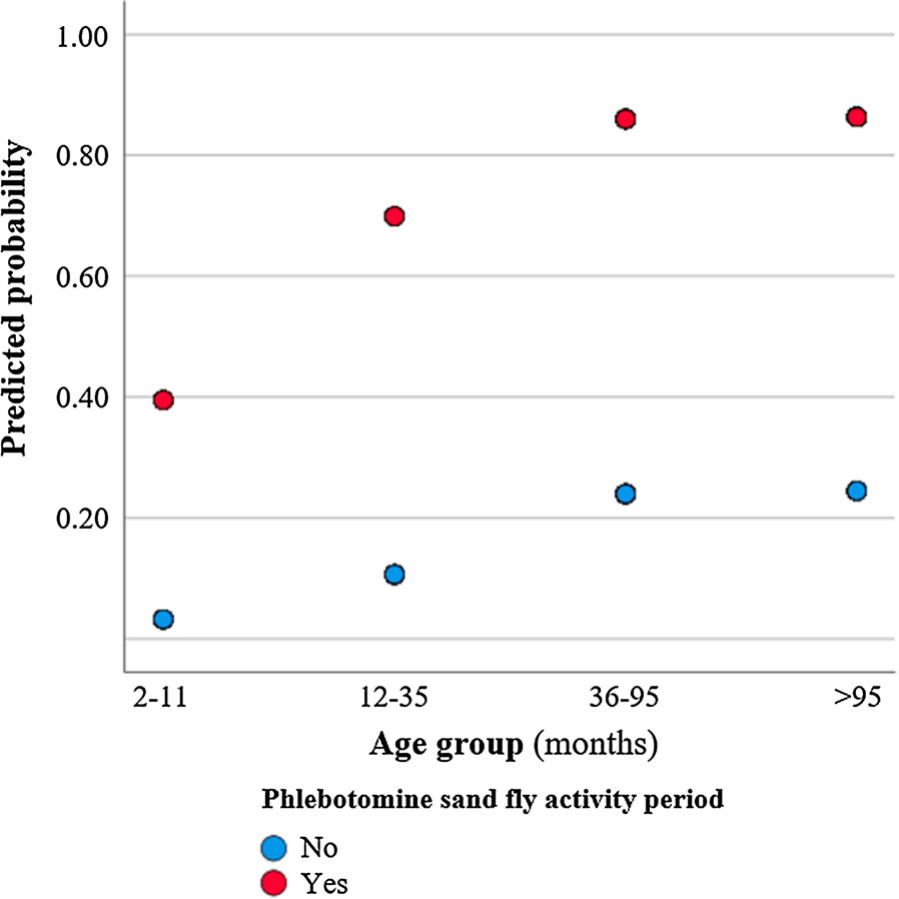
Predicted probability of the presence of antibodies against *Phlebotomus perniciosus* saliva related with cat age and phlebotomine sand fly activity period

Antibodies to *Phlebotomus perniciosus* saliva (cut-off ≥ 0.173) were detected in 167 (47.7%) sera (Table 1). One hundred and seven (73.8%) and 72 (35.8%) blood samples of domestic and stray cats, respectively, were collected during phlebotomine sand fly activity. There were significant differences between the ELISA result and the seven variables studied: (i) age group (*χ*^2^ = 38.335, *df* = 3, *P *< 0.001); (ii) fur length (*χ*^2^ = 6.229, *df* = 1, *P* = 0.043); (iii) lifestyle (*χ*^2^ = 31.806, *df* = 1, *P *< 0.001); (iv) region (*χ*^2^ = 14.246, *df* = 2, *P *< 0.001); (v) reproductive status (*χ*^2^ = 47.881, *df* = 1, *P *< 0.001); (vi) the use of acaricides/insecticides (*χ*^2^ = 20.516, *df* = 1, *P *< 0.001); and (vii) phlebotomine period activity (*χ*^2^ = 102.048, *df* = 1, *P *< 0.001). According to the multivariate logistic regression models, factors with a predicting effect on the presence of antibodies to *P. perniciosus* (Table 2) were age and phlebotomine activity period (Fig. 1). First, cats of 12–35 months had 3.56 higher odds (95% CI: 1.39–9.16; $\chi^{ 2}_{\text{Wald}}$ = 6.953, *df* = 1, *P* = 0.008) of presenting antibodies to *P. perniciosus* saliva than younger ones. This difference remained significant with higher magnitude when comparing young cats with those 36–95 months-old (OR = 9.43, 95% CI: 3.62–24.48; $\chi^{ 2}_{\text{Wald}}$ = 21.224, *df* = 1, *P *< 0.001) and those older than 95 months (OR = 9.68, 95% CI: 3.92–23.91; $\chi^{ 2}_{\text{Wald}}$ = 24.222, *df* = 1, *P *< 0.001). Secondly, sera collected during the period of phlebotomine sand fly seasonal activity exhibited nearly 19 times higher odds of having IgG antibody levels than those collected outside phlebotomine sand fly season (95% CI: 9.84–38.41; $\chi^{ 2}_{\text{Wald}}$ = 72.947, *df* = 1, *P *< 0.001).

**Table 1:** Prevalence of *Leishmania* (molecular and/or serological) and antibodies to *Phlebotomus perniciosus* saliva in cats from three regions of mainland Portugal

Variable/categories	Tested cats	Antibodies to *P. perniciosus* saliva			Antibodies to *Leishmania* and/or parasite DNA		
		Positive cats	95% CI	*P*-value	Positive cats	95% CI	*P*-value
Sex, *n* (%)	349			0.111 (*χ*^2^ = 2.535, *df* = 1)			0.468 (*χ*^2^ = 0.526, *df* = 1)
Female	191 (54.7)	84 (44.0)	37.1–51.1		16 (8.4)	5.2–13.2	
Male	158 (45.3)	83 (52.5)	44.8–60.2		10 (6.3)	3.5–11.3	
Age, median (IQR)	36 (12–96)	72 (24–121)		<0.001 (*Z* = -6.379)	28 (8–96)		0.301 (*Z* = -1.034)
Age group, *n* (%)	310			<0.001 (*χ*^2^ = 38.335, *df* = 3)			1.866 (*χ*^2^ = 1.866, *df* = 3)
2–11 months	70 (22.6)	16 (22.9)^a,b^	14.6–34.0		7 (10.0)	4.9–19.2	
12–35 months	67 (21.6)	25 (37.3)^c^	26.7–49.3		6 (9.0)	4.2–18.2	
36–95 months	85 (27.4)	39 (45.9)^a,d^	35.7–56.4		4 (4.7)	1.8–11.5	
More than 95 months	88 (28.4)	62 (70.5)^b,c,d^	60.2–79.0		6 (6.8)	3.2–14.1	
Reproductive status, *n* (%)	334			<0.001 (*χ*^2^ = 47.881, *df* = 1)			0.693 (*χ*^2^ = 0.156, *df* = 1)
Entire	216 (64.7)	72 (33.3)	27.4–39.9		14 (6.5)	3.9–10.6	
Neutered	118 (35.3)	86 (72.9)	64.2–80.1		9 (7.6)	4.1–13.9	
Breed, *n* (%)	347			0.811 (*χ*^2^ = 0.057 *df* = 1)			0.635^f^
Defined	18 (5.2)	9 (50.0)	29.0–71.0		2 (11.1)	3.1–32.8	
Mongrel	329 (94.8)	155 (47.1)	41.8–52.5		24 (7.3)	5.0–10.6	
Fur length, *n* (%)	349			0.013 (*χ*^2^ = 6.229, *df* = 1)			0.191^f^
Short	310 (88.8)	141 (45.5)	40.0–51.0		21 (6.8)	4.5–10.1	
Medium or long	39 (11.2)	26 (66.7)	51.0–79.4		5 (12.8)	5.6–26.7	
Lifestyle, *n* (%)	346			<0.001 (*χ*^2^ = 31.806, *df* = 1)			0.522 (*χ*^2^ = 0.411, *df* = 1)
Domestic	145 (41.9)	95 (65.5)	57.5–72.8		12 (8.3)	4.8–13.9	
Shelter/stray	201 (58.1)	70 (34.8)	28.6–41.6		13 (6.5)	3.8–10.8	
Region, *n* (%)	350			0.001 (*χ*^2^ = 14.246, *df* = 2)			0.467^f^
Centre	61 (17.4)	41 (67.2)^e^	54.7–77.7		5 (8.2)	3.6–17.8	
Lisbon metropolitan area	266 (76.0)	112 (42.1)^e^	36.3–48.1		18 (6.8)	5.6–26.7	
Algarve	23 (6.6)	14 (60.9)	40.8–77.8		3 (13.0)	4.5–32.1	
Other animals, *n* (%)	343			0.149 (*χ*^2^ = 2.082, *df* = 1)			0.197^f^
No	39 (11.4)	23 (59.0)	43.4–72.9		5 (12.8)	5.6–26.7	
Yes	304 (88.6)	142 (46.7)	41.2–52.3		21 (6.9)	4.6–10.3	
Ectoparasiticides, *n* (%)	332			<0.001 (*χ*^2^ = 20.516, *df* = 1)			0.147 (*χ*^2^ = 2.101, *df* = 1)
No	257 (77.4)	102 (39.7)	33.9–45.8		15 (5.8)	3.6–9.4	
Yes	75 (22.6)	52 (69.3)	58.2–78.6		8 (10.7)	5.5–19.7	
Clinical signs, *n* (%)	350			0.137 (*χ*^2^ = 2.212, *df* = 1			0.899 (*χ*^2^ = 0.899, *df* = 1)
No	252 (72.0)	114 (45.2)	39.2–51.4		19 (7.5)	4.9–11.5	
Yes	98 (28.0)	53 (54.1)	44.3–63.6		7 (7.1)	3.5–14.0	
Concomitant diseases, *n* (%)	181			0.185 (*χ*^2^ = 1.760, *df* = 1)			0.384 (*χ*^2^ = 0.759, *df* = 1)
No	99 (54.7)	62 (62.6)	52.8–71.5		11 (11.1)	6.3–18.8	
Yes	82 (45.3)	59 (72.0)	61.4–80.5		6 (7.3)	3.4–15.1	
Phlebotomine activity period, *n* (%)	350			<0.001 (*χ*^2^ = 102.048, *df* = 1)			0.156 (*χ*^2^ = 2.016, *df* = 1)
No	168 (48.0)	33 (19.6)	14.3–26.3		9 (5.4)	2.8–9.9	
Yes	182 (52.0)	134 (73.6)	66.8–79.5	0.398 (*χ*^2^ = 5.148, *df* = 5)	17 (9.3)	5.9–14.5	0.653 (*χ*^2^ = 3.308, *df* = 5)
May	25 (7.1)	20 (80.0)	60.9–91.1		3 (12.0)	2.5–31.2	
June	20 (5.7)	14 (70.0)	48.1–85.5		1 (5.0)	0.9–23.6	
July	22 (6.3)	20 (90.9)	72.2–97.5		2 (9.1)	2.5–27.8	
August	18 (5.1)	13 (72.2)	49.1–87.5		0 (0.0)	0.0–17.6	
September	24 (6.9)	17 (70.8)	50.8–85.1		2 (8.3)	2.3–25.9	
October	73 (20.9)	50 (68.5)	57.1–78.0		9 (12.3)	6.6–21.8	
Total, *n* (%)	350	167 (47.7)	42.5–52.9		26 (7.4)	5.1–10.7	

^a^*P* = 0.003 (*χ*^2^ = 8.832, *df* = 1)

^b^*P *< 0.001 (*χ*^2^ = 35.110, *df* = 1)

^c^*P *< 0.001 (*χ*^2^ = 16.860, *df* = 1)

^d^*P* = 0.001 (*χ*^2^ = 10.680, *df* = 1)

^e^*P *< 0.001 (*χ*^2^ = 12.530, *df* = 1)

^f^Fisher’s exact test or Freeman-Halton test

*Abbreviations*: CI, confidence interval; IQR, interquartile interval (quartile 1 - quartile 3)

**Table 2:** Presence of antibodies against *Phlebotomus perniciosus* saliva: odds-ratios, 95% confidence intervals and significances, obtained by simple (OR crude) and multiple (OR adjusted) logistic regression models

Variable/categories	OR crude	95% CI	*P*-value	OR adjusted	95% CI	*P*-value
Sex^b^						
Female^a^						
Male	1.41	0.92–2.15	0.112 ($\chi^{ 2}_{\text{Wald}}$ = 2.528, *df* = 1)			
Age group						
2–11 months^a^			<0.001 ($\chi^{ 2}_{\text{Wald}}$ = 35.190, *df* = 3)			<0.001 ($\chi^{ 2}_{\text{Wald}}$ = 29.553, *df* = 3)
12–35 months	2.01	0.95–4.24	0.067 ($\chi^{ 2}_{\text{Wald}}$ = 3.360, *df* = 1)	3.56	1.39–9.16	0.008 ($\chi^{ 2}_{\text{Wald}}$ = 6.953, *df* = 1)
36–95 months	2.86	1.42–5.78	0.003 ($\chi^{ 2}_{\text{Wald}}$ = 8.608, *df* = 1)	9.43	3.62–24.48	<0.001 ($\chi^{ 2}_{\text{Wald}}$ = 21.224, *df* = 1)
More than 95 months	8.05	3.91–16.56	<0.001 ($\chi^{ 2}_{\text{Wald}}$ = 32.070, *df* = 1)	9.68	3.92–23.91	<0.001 ($\chi^{ 2}_{\text{Wald}}$ = 24.222, *df* = 1)
Reproductive status^b^						
Entire^a^						
Neutered	5.38	3.28–8.82	<0.001 ($\chi^{ 2}_{\text{Wald}}$ = 44.393, *df* = 1)			
Breed^b^						
Defined^a^						
Mongrel	0.89	0.35–2.30	0.811 ($\chi^{ 2}_{\text{Wald}}$ = 0.057, *df* = 1)			
Fur length^b^						
Short^a^						
Medium or long	2.40	1.19–4.84	0.015 ($\chi^{ 2}_{\text{Wald}}$ = 5.953, *df* = 1)			
Lifestyle^b^						
Domestic^a^						
Shelter/stray	0.28	0.180–0.44	<0.001 ($\chi^{ 2}_{\text{Wald}}$ = 30.684, *df* = 1)			
Region^b^						
Centre^a^			0.001 ($\chi^{ 2}_{\text{Wald}}$ = 13.693, *df* = 2)			
Lisbon metropolitan area	0.36	0.20–0.64	0.001 ($\chi^{ 2}_{\text{Wald}}$ = 11.957, *df* = 1)			
Algarve	0.76	0.28–2.05	0.586 ($\chi^{ 2}_{\text{Wald}}$ = 0.297, *df* = 1)			
Other animals^b^						
No^a^						
Yes	0.61	0.31–1.20	0.152 ($\chi^{ 2}_{\text{Wald}}$ = 2.053, *df* = 1)			
Ectoparasiticides^b^						
No^a^						
Yes	3.44	1.98–5.96	<0.001 ($\chi^{ 2}_{\text{Wald}}$ = 19.290, *df* = 1)			
Clinical signs^b^						
No^a^						
Yes	1.43	0.89–2.28	0.138 ($\chi^{ 2}_{\text{Wald}}$ = 2.203, *df* = 1)			
Concomitant diseases^b^						
No^a^						
Yes	1.53	0.82–2.88	0.186 ($\chi^{ 2}_{\text{Wald}}$ = 1.751, *df* = 1)			
Phlebotomine activity period^b^						
No^a^						
Yes	11.42	6.90–18.90	<0.001 ($\chi^{ 2}_{\text{Wald}}$ = 89.858, *df* = 1)	19.44	9.84–38.41	<0.001 ($\chi^{ 2}_{\text{Wald}}$ = 72.947, *df* = 1)

^a^Reference category

^b^Variable that did not have a statistically significant association in the fitted model

*Abbreviations*: OR, odds ratio; CI, confidence interval

*Leishmania* infection was detected in 26 cats (7.7%): *Leishmania* spp. DNA was detected using a set of general primers that target *SSU* rRNA in the blood samples of 24 (6.9%) cats, while antibodies to *L. infantum* were detected in three (0.9%) sera.

Only one cat was positive to *Leishmania* by both techniques. No significant differences were detected in positivity to *L. infantum* among all the variables/categories studied (Table 1).

*Leishmania* DNA or specific antibodies to the parasite were detected in 18 cats seropositive to phlebotomine sand fly saliva. Of these 18 cats, all but one had a blood sample taken during phlebotomine sand fly activity. Cats presenting IgG antibodies to *P. perniciosus* had significantly higher risk ($\chi^{ 2}_{\text{Wald}}$ = 4.893, *df* = 1, *P* = 0.027; OR = 2.64, 95% CI: 1.12–6.25) of being infected with *Leishmania* (Table 3).

**Table 3:** Association between the presence of antibodies to *Phlebotomus perniciosus* saliva and a serological and/or molecular positive result for *Leishmania*

Variable/categories	Antibodies to *Leishmania* and/or parasite DNA						
	Tested cats, *n* (%)	Positive cats, *n* (%)	Chi-square test		Simple logistic regression model		
			95% CI	*P*-value	OR	95% CI	*P*-value
*Phlebotomus perniciosus* saliva	350			0.022 (*χ*^2^ = 5.212, *df* = 1)			
Seronegative^a^	183 (52.3)	8 (4.4)	2.2–8.4				
Seropositive	167 (47.7)	18 (10.8)	6.9–16.4		2.64	1.12–6.25	0.027 ($\chi^{ 2}_{\text{Wald}}$ = 4.893, *df* = 1)

^a^Reference category

*Abbreviations*: CI, confidence interval; OR, odds ratio

## Discussion

To our knowledge, this study describes for the first time feline antibody response against *P. perniciosus* saliva in cats naturally exposed to phlebotomine sand flies. The detection of antibodies to *P. perniciosus* in 47.7% of sera shows that cats are frequently bitten by this sand fly, which is the most abundant *Phlebotomus* species in the three studied regions [13, 26]. The presence of IgGs in 73.6% of sera tested during phlebotomine activity period corroborates the results obtained in dogs from the Lisbon metropolitan area, where antibodies to *P. perniciosus* SGH were detected in 181 (75.1%) out of 241 animals at the beginning of phlebotomine sand fly activity (i.e. May) and in 209 (86.7%) out of 241 at the end of phlebotomine sand fly season (i.e. October) [27].

Previous studies have demonstrated that canine antibodies to phlebotomine sand fly-saliva correlate with biting intensity, fluctuate within phlebotomine sand fly season and decline significantly after the end of the biting season [6, 7, 12], emphasizing their usefulness as biomarkers for evaluating the exposure to phlebotomine sand flies and efficacy of vector control campaigns [4, 28].

In the present study, two non-casual associations were observed in the univariate analysis, namely the presence of a higher percentage of *P. perniciosus* antibodies in domestic and treated cats with ectoparasiticides than in stray and untreated cats, respectively. The reasons for these non-casual associations can be explained with the fact that most (73.8%; 107/145) of the blood samples of the domestic cats with access to the outdoors were taken during the exposure period to phlebotomine sand fly bites, while only 35.8% (72/201) of stray cats were sampled during phlebotomine sand fly season activity. On the other hand, the fact that cats treated with ectoparasiticides did not show a lower prevalence of positivity to *P. perniciosus* saliva than untreated cats was not entirely surprising because the only repellents effective against phlebotomine sand flies, the pyrethroids, are toxic to cats, with the exception of flumethrin. However, the application of imidacloprid/flumethrin collars in cats is still quite low in Portugal [29]. Nevertheless, and despite the lack of repellent effect of the most common ectoparasiticides applied to cats, they can potentially prevent parasite transmission from treated animals to other vertebrate hosts.

However, based on multivariate analysis, the presence of *P. perniciosus* antibodies in the peripheral blood of cats was neither associated with lifestyle nor with the use of ectoparasiticides, suggesting that these two variables are confounders. These results reinforce the importance of multivariate analysis in addressing confounding in epidemiological studies [30]. Based on this analysis, an association between IgG positivity and phlebotomine sand fly seasonal activity was observed in the present study, being significantly higher between May and October, than during winter months, when phlebotomine sand flies are inactive, suggesting that feline antibodies to saliva are relatively short-living. Unfortunately, no data are available for cats regarding the kinetics of specific antibodies to phlebotomine sand fly saliva or their correlation with the number of phlebotomine sand fly bites; therefore, it is not possible to precisely correlate feline antibodies against sand fly SGH and the seasonal abundance of *P. perniciosus.*

In cats, the antibody levels to *P. perniciosus* saliva were significantly increased with age group, suggesting accumulative exposure of older animals to sand fly bites. A similar positive correlation was repeatedly demonstrated in dogs [8] which is probably related to the re-exposure of vertebrate hosts to phlebotomine sand flies following antigenic priming in the previous seasons. Interestingly, cats presenting antibodies to saliva were significantly more at risk of being positive to *Leishmania* infection. Whether saliva antigens could be used as biomarkers for *Leishmania* infection remains controversial, since both positive [7, 10, 11] and negative [6] associations between anti-*P. perniciosus* SGH antibodies and active *L. infantum* infection have been observed in dogs from endemic areas of leishmaniosis [4].

Regarding *Leishmania* infection, antibodies to the parasite or its DNA were detected in 26 cats (7.7%). The positivity of detection of *Leishmania* DNA (6.9%) was higher than the 0.3% previously obtained in the north and centre of Portugal [17], but lower than the one (9.9%) obtained in the south of the country [19], reinforcing that the rate of *Leishmania* infection is dynamic over time, depending on the density of proven vector population and on the number of infected vertebrate hosts.

Antibodies to *Leishmania* were detected by IFAT in 3 cats (0.9%), which is also in agreement with previous studies performed in domestic and stray cats from the metropolitan Lisbon area [18, 20] but lower than the 3.8% of seropositivity obtained in cats from the Algarve region [31]. This strengthens the assumption that IFAT might not be sensitive enough to detect *Leishmania* infection in cats, or at least in those subclinically infected [2, 32].

## Conclusions

To our knowledge, this is the first study demonstrating the development of anti-sand fly saliva antibodies in cats. Due to the potential role of this animal species in sustaining and spreading *L. infantum* infection, the evaluation of the contact of cats with the vector is important in the development of prophylactic measures directed to cats with the aim of reducing the prevalence of infection in an endemic area. Further studies are needed to evaluate if there is a correlation between the number of phlebotomine sand fly bites and the dynamics of antibody production and if the use of imidacloprid/flumethrin collars reduces the frequency of *P. perniciosus* bites and *L. infantum* positivity in cats.

## Abbreviations

CanL: Canine leishmaniosis
CI: Confidence intervals
ELISA: Enzyme-linked immunosorbent assay
IFAT: immunofluorescence antibody test
L: *Leishmania*
OR: Odds ratio
SGH: salivary gland homogenate

## References

[1] Pennisi M-G, Cardoso L, Baneth G, Bourdeau P, Koutinas A, Miró G (2015). LeishVet update and recommendations on feline leishmaniosis. Parasit Vectors..

[2] Maia C, Campino L (2011). Can domestic cats be considered reservoir hosts of zoonotic leishmaniasis?. Trends Parasitol..

[3] Maia C, Dantas-Torres F, Campino L, Bruschi F, Gradoni L (2018). Parasite biology: the reservoir hosts. The leishmaniases: old neglected tropical diseases.

[4] Lestinova T, Rohousova I, Sima M, de Oliveira CI, Volf P (2017). Insights into the sand fly saliva: blood-feeding and immune interactions between sand flies, hosts, and *Leishmania*. PLoS Negl Trop Dis..

[5] Teixeira C, Gomes R, Collin N, Reynoso D, Jochim R, Oliveira F (2010). Discovery of markers of exposure specific to bites of *Lutzomyia longipalpis*, the vector of *Leishmania infantum chagasi* in Latin America. PLoS Negl Trop Dis..

[6] Vlkova M, Rohousova I, Drahota J, Stanneck D, Kruedewagen EM, Mencke N (2011). Canine antibody response to *Phlebotomus perniciosus* bites negatively correlates with the risk of *Leishmania infantum* transmission. PLoS Negl Trop Dis..

[7] Kostalova T, Lestinova T, Sumova P, Vlkova M, Rohousova I, Berriatua E (2015). Canine antibodies against salivary recombinant proteins of *Phlebotomus perniciosus*: a longitudinal study in an endemic focus of canine leishmaniasis. PLoS Negl Trop Dis..

[8] Kostalova T, Lestinova T, Maia C, Sumova P, Vlkova M, Willen L (2017). The recombinant protein rSP03B is a valid antigen for screening dog exposure to *Phlebotomus perniciosus* across foci of canine leishmaniasis. Med Vet Entomol..

[9] Solcà MS, Andrade BB, Abbehusen MMC, Teixeira CR, Khouri R, Valenzuela JG (2016). Circulating biomarkers of immune activation, oxidative stress and inflammation characterize severe canine visceral leishmaniasis. Sci Rep..

[10] Quinnell RJ, Soremekun S, Bates PA, Rogers ME, Garcez LM, Courtenay O (2018). Antibody response to sand fly saliva is a marker of transmission intensity but not disease progression in dogs naturally infected with *Leishmania infantum*. Parasit Vectors..

[11] Velez R, Spitzova T, Domenech E, Willen L, Cairó J, Volf P (2018). Seasonal dynamics of canine antibody response to *Phlebotomus perniciosus* saliva in an endemic area of *Leishmania infantum*. Parasit Vectors..

[12] Hostomska J, Rohousova I, Volfova V, Stanneck D, Mencke N, Volf P (2008). Kinetics of canine antibody response to saliva of the sand fly *Lutzomyia longipalpis*. Vector Borne Zoonotic Dis..

[13] Alten B, Maia C, Afonso MO, Campino L, Jiménez M, González E (2016). Seasonal dynamics of phlebotomine sand fly species proven vectors of Mediterranean leishmaniasis caused by *Leishmania infantum*. PLoS Negl Trop Dis..

[14] Cortes S, Vaz Y, Neves R, Maia C, Cardoso L, Campino L (2012). Risk factors for canine leishmaniasis in an endemic Mediterranean region. Vet Parasitol..

[15] Alvar J, Vélez ID, Bern C, Herrero M, Desjeux P, Cano J (2012). Leishmaniasis worldwide and global estimates of its incidence. PLoS One..

[16] Maia C, Sousa C, Ramos C, Cristóvão JM, Faísca P, Campino L (2015). First case of feline leishmaniosis caused by *Leishmania infantum* genotype E in a cat with a concurrent nasal squamous cell carcinoma. JFMS Open Rep..

[17] Vilhena H, Martinez-Díaz VL, Cardoso L, Vieira L, Altet L, Francino O (2013). Feline vector-borne pathogens in the north and centre of Portugal. Parasit Vectors..

[18] Maia C, Gomes J, Cristóvão J, Nunes M, Martins A, Rebêlo E (2010). Feline *Leishmania* infection in a canine leishmaniasis endemic region, Portugal. Vet Parasitol..

[19] Maia C, Ramos C, Coimbra M, Bastos F, Martins A, Pinto P (2014). Bacterial and protozoal agents of feline vector-borne diseases in domestic and stray cats from southern Portugal. Parasit Vectors..

[20] Maia C, Nunes M, Campino L (2008). Importance of cats in zoonotic leishmaniasis in Portugal. Vector-Borne Zoonotic Dis..

[21] Maia C, Dionísio L, Afonso MO, Neto L, Cristóvão JM, Campino L (2013). *Leishmania* infection and host-blood feeding preferences of phlebotomine sandflies and canine leishmaniasis in an endemic European area, the Algarve Region in Portugal. Mem Inst Oswaldo Cruz..

[22] Branco S, Alves-Pires C, Maia C, Cortes S, Cristovão JMS, Gonçalves L (2013). Entomological and ecological studies in a new potential zoonotic leishmaniasis focus in Torres Novas municipality, Central Region, Portugal. Acta Trop..

[23] Volf P, Volfova V (2011). Establishment and maintenance of sand fly colonies. J Vector Ecol..

[24] Maia C, Nunes M, Cristóvão J, Campino L (2010). Experimental canine leishmaniasis: clinical, parasitological and serological follow-up. Acta Trop..

[25] Cruz I, Cañavate C, Rubio JM, Morales MA, Chicharro C, Laguna F (2002). A nested polymerase chain reaction (Ln-PCR) for diagnosing and monitoring *Leishmania infantum* infection in patients co-infected with human immunodeficiency virus. Trans R Soc Trop Med Hyg..

[26] Pereira M (2008). Identificação e densidade das espécies flebotomínicas no concelho de Coimbra: vectores de leishmanioses.

[27] Cristóvão J (2015). Monitorização do risco de exposição à leishmaniose zoonótica.

[28] Brodskyn CI, Kamhawi S (2018). Biomarkers for zoonotic visceral leishmaniasis in Latin America. Front Cell Infect Microbiol..

[29] Pereira A, Martins Â, Brancal H, Vilhena H, Silva P, Pimenta P (2016). Parasitic zoonoses associated with dogs and cats: a survey of Portuguese pet owners’ awareness and deworming practices. Parasit Vectors..

[30] Jager KJ, Zoccali C, MacLeod A, Dekker FW (2008). Confounding: what it is and how to deal with it. Kidney Int..

[31] Maia C, Ramos C, Coimbra M, Cardoso L, Campino L (2015). Prevalence of *Dirofilaria immitis* antigen and antibodies to *Leishmania infantum* in cats from southern Portugal. Parasitol Int..

[32] Chatzis MK, Leontides L, Athanasiou LV, Papadopoulos E, Kasabalis D, Mylonakis M (2014). Evaluation of indirect immunofluorescence antibody test and enzyme-linked immunosorbent assay for the diagnosis of infection by *Leishmania infantum* in clinically normal and sick cats. Exp Parasitol..

